# Engineering considerations for practical lithium–air electrolytes†

**DOI:** 10.1039/d3fd00091e

**Published:** 2023-08-01

**Authors:** James H. J. Ellison, Clare P. Grey

**Affiliations:** a Yusuf Hamied Department of Chemistry, University of Cambridge Cambridge UK cpg27@cam.ac.uk

## Abstract

Lithium–air batteries promise exceptional energy density while avoiding the use of transition metals in their cathodes, however, their practical adoption is currently held back by their short lifetimes. These short lifetimes are largely caused by electrolyte breakdown, but despite extensive searching, an electrolyte resistant to breakdown has yet to be found. This paper considers the requirements placed on an electrolyte for it to be considered usable in a practical cell. We go on to examine ways, through judicious cell design, of relaxing these requirements to allow for a broader range of compounds to be considered. We conclude by suggesting types of molecules that could be explored for future cells. With this work, we aim to broaden the scope of future searches for electrolytes and inform new cell design.

## Abstract 2

We consider the requirements to be placed on an electrolyte for it be used in a practical lithium–air battery. Ways to ease these requirements by refining cell design and improving transport as well as motifs for future electrolytes are discussed.

## Graphical abstract

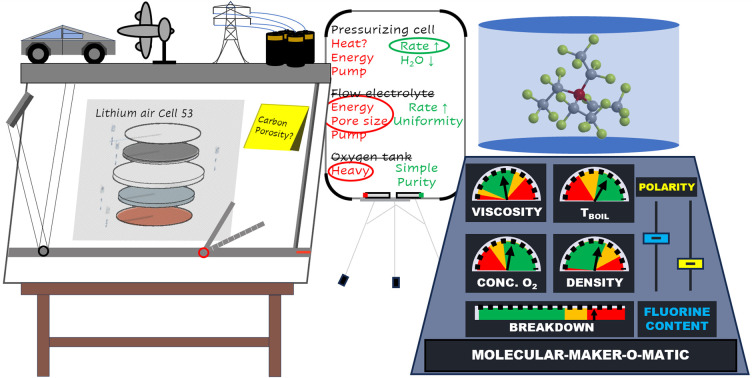

## Introduction

Lithium–air batteries (LABs) are a promising technology for energy storage in vehicles and mobile applications due to a combination of their exceptional energy density and the lack of transition metals in their cathodes. However, their short lifetimes have hindered their widespread adoption. Principally this is due to the steady build-up of degradation products on the carbon cathode surface. This is primarily caused by the breakdown of the electrolyte due to its oxidation by reactive oxygen species formed in the cell. Despite numerous attempts to find a viable electrolyte for lithium–air batteries that is resistant to breakdown while enabling successful cell operation, a workable solution has yet to be discovered.

Previous searches for new electrolytes have been conducted for Li–air electrolytes, trying to find solvents with bounds placed on factors such as viscosity, boiling point, ionisation potential and p*K*_a_, among others.^1,2^ These searches, unfortunately, met with limited success, with the authors noting that it was hard to fulfil all the requirements simultaneously^3^ and that it will be necessary to explore beyond the known chemical space.^1^

Other works have focused on system analyses to assess the likely energy density^4,5^ and costs achievable^4^ with LABs. However, these works have typically not considered ionic and molecular transport properties in the electrolyte and their implications for the electrolyte selection.

This paper explores ways to engineer the battery to relax the requirements on the electrolyte, while still enabling a high-performance battery. To do this, we first define what we consider a practical battery. We then discuss the implications of these requirements on the properties required for the electrolyte, and ways to widen the bounds on these properties. We conclude by presenting a guide to engineering a battery for high performance, as well as the newly relaxed requirements on the electrolyte to inform future searches.

Throughout this paper, we will make a number of assumptions about what is reasonable performance for the cell and its components. We endeavour to motivate and justify these choices, but they still represent only one possible example of a LAB. For example, we do not consider a solid electrolyte between the cathode and anode, or cycling *via* LiOH rather than Li_2_O_2_. Some of the discussion will translate to other metal–air batteries, although we do not explore these batteries thoroughly. Still, we try to generalise the discussion to cover a broad range of situations. We also make available an Excel spreadsheet as ESI† so that others can explore different assumptions and conditions to investigate the other incarnations of a LAB.

## Methodology

We start by setting out the equations, notation and assumptions that we will be using to inform our later discussion of solvent properties.

### Specific energy

Specific energy can simply be calculated by summing the specific energy, *e*, of each component multiplied by the material's mass fraction (eqn (1), where *M* denotes mass). In a similar manner, the energy density, *ρ*, (energy per unit volume) is calculated by summing the energy densities times the volume fraction (eqn (2), where *V* denotes volume) 1
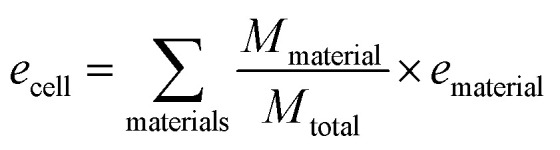
2
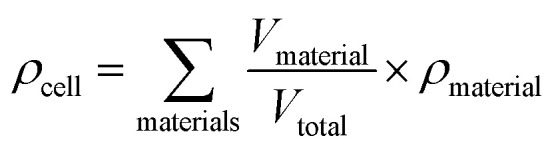

3 *e*_material_ = *V*_Li^+^*vs.* ref_ × specific capacity

4 *ρ*_material_ = *V*_Li^+^*vs.* ref_ × capacity density

where *V*_Li^+^*vs.* ref_ is the potential of the material *vs.* some reference electrode (for example, the standard hydrogen electrode).

This definition of the specific energy of each material is dependant on the voltage, which has some reference point; this can be arbitrarily chosen (for a full discussion, see the ESI†).

### Mass transport

Oxygen diffusion is considered to obey Fick's first law:5
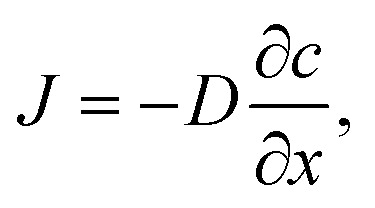
where *J* is the diffusion flux, *D* is the diffusivity, *c* is the concentration of oxygen, and *x* is the distance being considered. We then assume uniform consumption of O_2_ across the cell:6
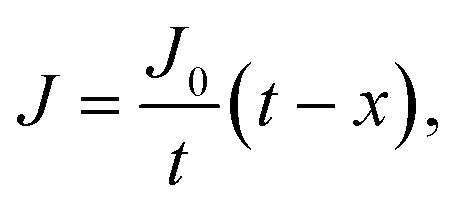
where *J*_0_ is the surface flux of O_2_, and *t* is the depth into the cell. Combining eqn (5) and eqn (6) gives7
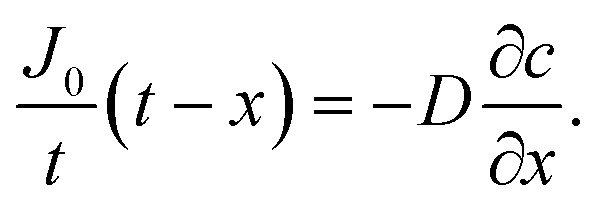


By integrating this equation, we can find the required diffusivity and surface concentration to ensure all areas of the cell have greater than zero O_2_ concentration8
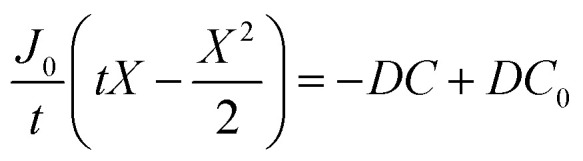
9
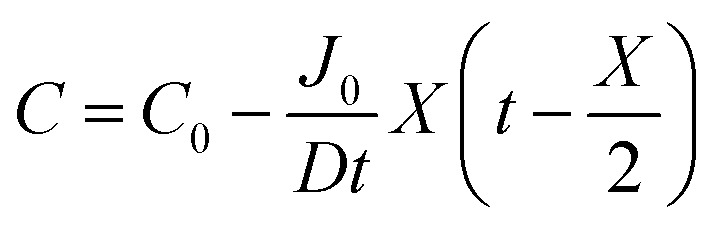
10
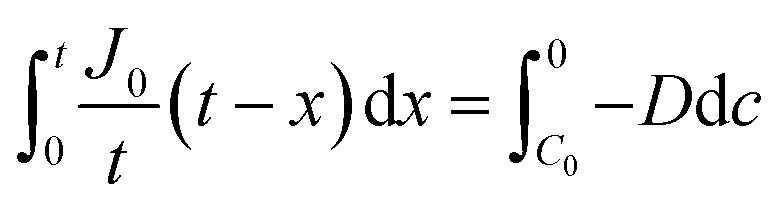
11
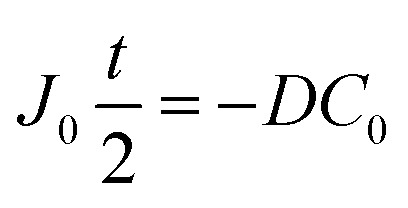
where *C* is the concentration of oxygen at the position of interest, *X* is the distance of the position of interest from the electrolyte/gas interface. *C*_0_ is the equilibrium concentration of oxygen in the electrolyte at the electrolyte/gas interface.

To account for the porosity (*ε*) and tortuosity (*τ*), we calculate *D*_eff_ (the effective diffusivity) as follows:12
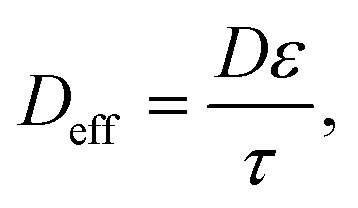
with tortuosity calculated by the Maxwell model.^6^ We note more complex and potentially more accurate models exist, but for the relatively low porosity considered here, the Maxwell model is considered to be sufficient.13
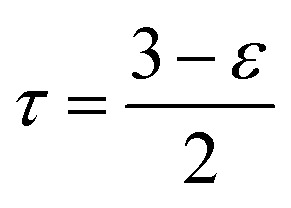


We model the pressure drop (*P*) across the cell using Darcy's law:14
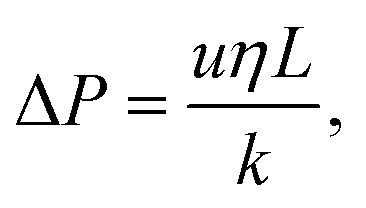
where *u* is the velocity of the fluid, *η* is the viscosity, and *L* is the length over which the fluid flows. The permeability (*k*) is calculated by:^7^15
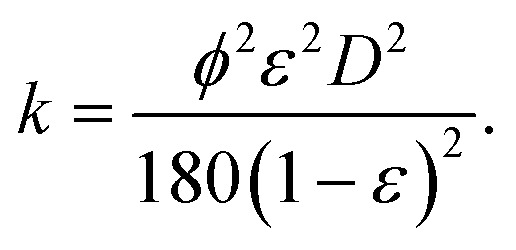


Here we have assumed that the sphericity of the particles (*ϕ*) is always 1 and defined by an average particle diameter *D*. Combining eqn (14) and (15) gives the Kozeny–Carman equation.

### Pressure vessel

The mass (*M*) of a thin-walled pressure vessel is given by:^8^16
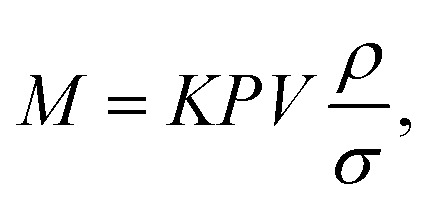
where *k* is a constant dependant on the shape of the pressure vessel, and in the best case of a sphere is 
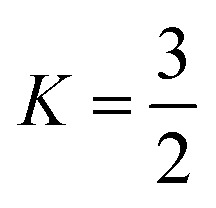
. The other variables are the pressure difference (*P*), the volume of gas (*V*), the density of structural material (*ρ*), and the tensile strength (*σ*). We also introduce a safety factor (S.F.), which is the amount by which the theoretical tensile yield strength of a material is reduced to provide a safety margin, and for which a value of 2 is often used for pressure vessels.^9^ Thus, for a given temperature and assuming atmospheric pressure is negligible in comparison to the stored gas pressure, the mass of the tank required to store a gas is directly proportional to the amount of gas stored in it. This is shown by assuming our gas is ideal and follows:

17 *PV* = *nRT*,

where *n* is the number of moles, *R* is the ideal gas constant, and *T* is temperature.

Thus, in our case, with 
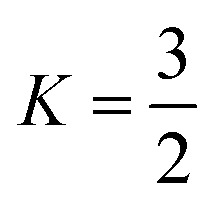
, *V* = *nRT*/*P*, and *σ* is reduced by the S.F.:18
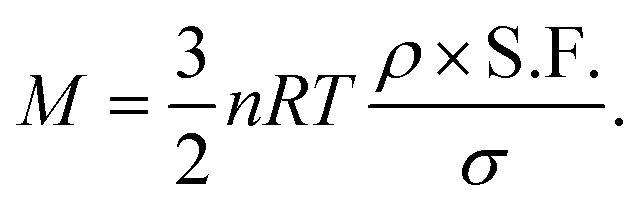


### Evaporation rate

We start with the Clausius–Clapeyron equation19
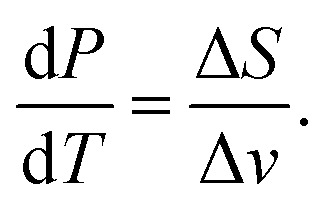
Where Δ*S* is the entropy change of the phase transition and Δ*v* the change in molar volume.

If we assume an ideal gas and neglect the volume of the liquid phase, we can rewrite this as:20
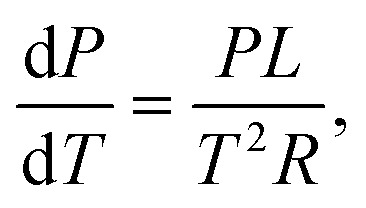
where *L* is the specific latent heat of vaporisation. Integrating this between two temperatures we get21
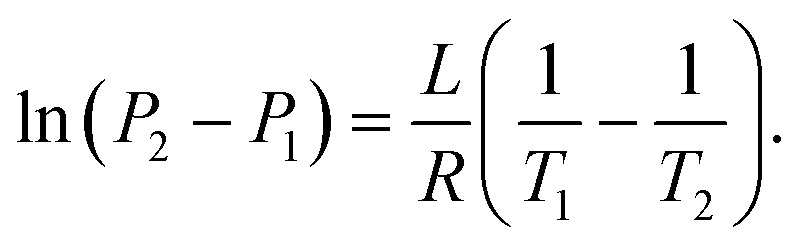


By considering one of those temperatures as the boiling point, we can calculate the vapour pressure (*P*_2_) at a given temperature (*T*_2_).22
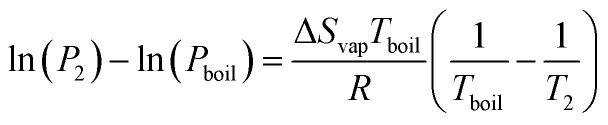


We here assume Trouton's rule is valid:

23 Δ*S*_vap_ ≈ 10.5*R*,

which finally gives us24
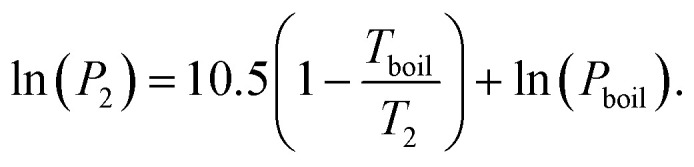


We also assume that the moles of solvent lost in evaporation is proportional to the product of its vapour pressure and its diffusivity. The diffusivity of the solvent is taken, in our cell, to be 10% of the oxygen diffusivity. The value is determined from extrapolating based on the molecular weight of tetraglyme following hydrocarbon diffusivity data.^10^

### Hansen solubility parameters

The Hansen solubility parameters are dispersion (*δ*_D_), polar (*δ*_P_) and H-bonding (*δ*_H_) terms, which are related to the cohesive energy (*E*) and in turn to the enthalpy of vaporisation (*H*_v_).25
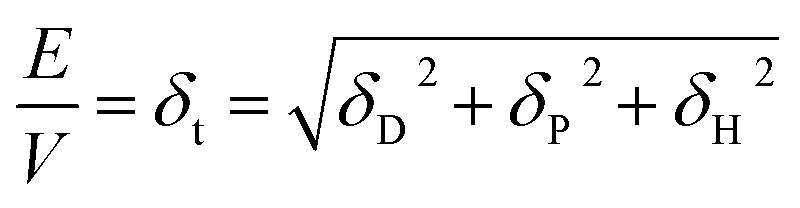

26 *H*_v_ = *E* − *RT*

where *V* is molar volume, *δ*_t_ is the total Hansen solubility parameter.^11^

The solubility of a substance in a solvent can be estimated by the distance in the Hansen parameter space between their solubility parameters (*R*_H_), with the dispersion term receiving a higher weighting.27
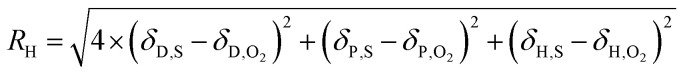


Others^12^ have related this distance in the specific case of O_2_ to its molar fraction in the solvent *via*:

28 log_10_(molar fraction O_2_) = 0.0889*R*_H_ − 1.1.

### Diffusion

We consider O_2_ to diffuse *via* the Stokes–Einstein equation29
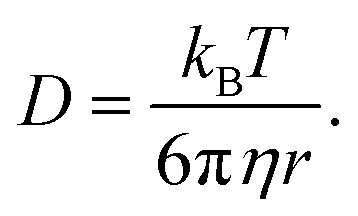
where *r* is the affective (spherical) radius of the molecule of interest.

The relationship between viscosity and boiling point of a solvent is fairly weak and can lead to sizable errors. However, it can be approximated as^13^30
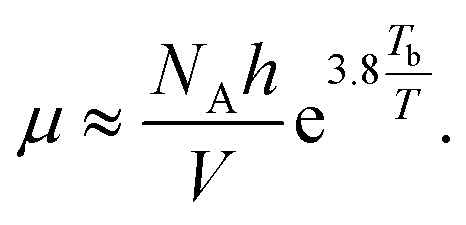
where *μ* is the viscosity, *N*_A_ is Avagardo’s number, *h* is Planck’s constant, and *T*_b_ the boiling point of the liquid.

### Ionic transport

The Sand's time (*t*_sand_) is defined as:31
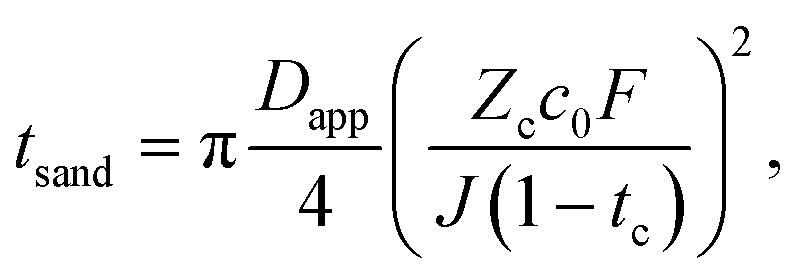
where the effective diffusion constant of Li^+^ ions in the electrolyte (*D*_app_), the charge of the cation (*Z*_c_) in our case is +1, and the other terms include its transference number (*t*_c_), the salt concentration (*c*_0_), Faraday constant (*F*) and current density (*J*). Typically, the Sand's time is considered to refer to the depletion of lithium ions at the lithium metal anode during charging. However, in the case of lithium–air batteries, one could in principle deplete areas of the cathode of lithium ions during discharge, as the lithium ions are precipitated from solution to form Li_2_O_2_. This would either result in a rapid charge build-up from the formation of O_2_^2−^, preventing further discharge, or some form of degradation to balance this charge formation. Neither of these would be desirable.

We also consider the limiting current *J*_lim_ below which the sand time is effectively infinite.32
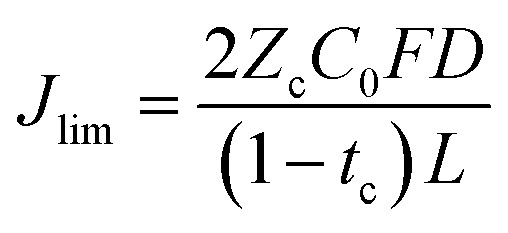
where μ is the viscosity, *N*_A_ is Avagardo's number, *h* is Planck's constant, and *T*_b_ the boiling point of the liquid.

### Turbine

We consider the work required to perform adiabatic compression of a gas (*W*_adi,comp_):33
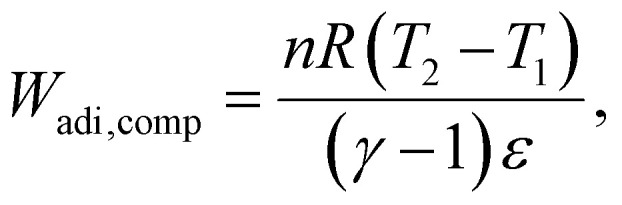
where *γ* is the ratio of specific heats and *ε* is the turbine efficiency. The work on expansion is then given by:34
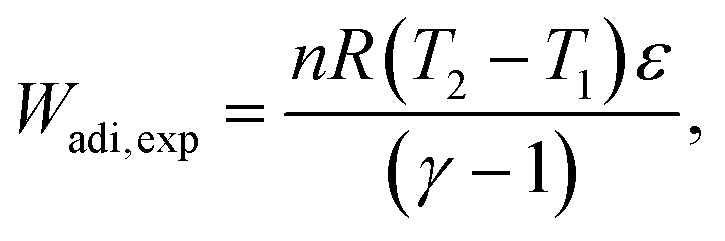
and this gives an outlet temperature (*T*_2_)35
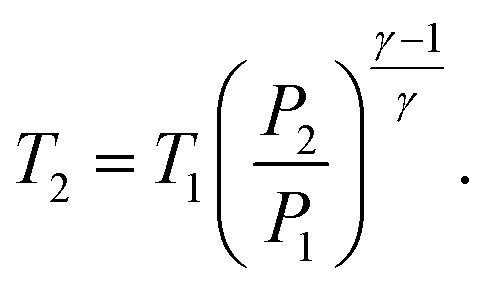


The work done by an isothermal compression (*W*_iso,comp_) is calculated by36
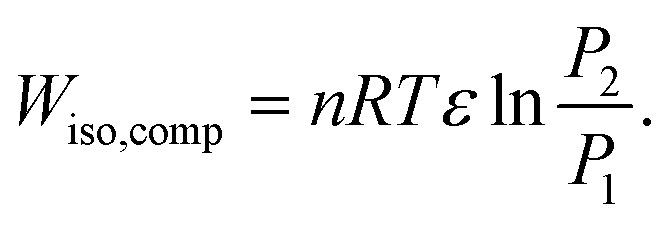


For an ideal gas, this is also equal to the heat output.

## Results

### Requirements for a practical battery

To define a practical battery, we will consider three possible use cases: a battery pack for an electric vehicle, for grid storage and for a high-altitude long duration solar-powered drone. The former two are selected as they are critical to enabling the energy transition. We include the latter case as a potential early adopter of Li–air batteries, with slow (12 hours) discharge/charge cycles, tolerance to short lifetimes (tens of days and hence tens of cycles), operating in dry air and requiring high energy density. We do note, however, that Li–air batteries may not be best suited to grid storage, as the grid highly prizes lifetimes, an area in which LABs currently struggle. These are by no means the only use cases, but are taken as examples.

Clearly, other requirements beyond Table 1, for example, cost, safety, toxicity and operating conditions, exist in these use cases. At points, we will consider these, but a full consideration of them lies beyond the scope of this discussion. We summarise the targets for our cell as 700 Wh kg^−1^, 700 Wh L^−1^, 80% efficiency and a 4 h charge/discharge cycle, and attempt to maximise the lifetime given these requirements.

**Table 1:** Requirements that need to be met for the cell to be considered practical. These are chosen to be minimal requirements to enable entry into the respective markets. A full discussion of why we consider these practical values can be found in the ESI.†

	Grid	Car	Drone
Specific energy (Wh kg^−1^)	—	700+	700+
Energy density (Wh L^−1^)	—	700+	700+
Energy efficiency	80%	75%	60%
Cycle life (to 80%)	3000?	600	12
Charge/discharge time (h)	4/4+	<4/4	12/12

We highlight that the efficiency of the battery is strongly related to the overpotential, on both charge and discharge, of the cell. This is strongly influenced by the electrolyte and the mechanism for Li_2_O_2_ formation and removal. This is discussed only tangentially in this work and has been discussed more thoroughly elsewhere.^14,15^ For our purposes, we consider that if we can minimise breakdown, thereby maximising coulombic efficiency, and prevent transport from becoming a limiting factor, thus improving voltage efficiency, this should enable the cell to operate with a high energy efficiency.

### Cell geometry

We start by considering the specific energy (*e*), that is the energy per unit mass of the battery, and the implications of meeting this target. We consider this first as the requirements placed here on component sizes have significant effects on other requirements. It is also an easy parameter to calculate and so provides a firm foundation to build on.

Our example cell is considered at the individual cell level. We thus neglect the mass of any cooling, intercell connectors or state-of-charge monitoring equipment in our calculations.

When considering anode materials for a LAB, we see (Table 2 in the ESI†) that lithiated graphite with a high-performance carbon could in principle meet the energy density targets; however, even with optimistic projections for other components, it seems unlikely a realistic cell would be created with >700 Wh kg^−1^ and lithiated graphite. Lithiated silicon is not ruled out. However, our discussion will consider lithium metal as it is by far the most common anode considered for lithium–air batteries and it has been assumed that the lithium-ion competition will be using lithium metal. Additionally, other than solid electrolyte interphase (SEI) considerations, lithiated silicon could easily be swapped in for lithium metal if that is preferred.

Clearly inert materials, such as the casing, carbon and, most relevant to us, electrolytes, have no specific energy, although without these the cell would not function. Thus, these components' mass fraction should be minimised to maximise the specific energy of the cell. However, our aim is to place the most lenient restrictions on our electrolyte as possible so we do not constrain the electrolyte's density at this point.

Minimising the mass of other components comes with side effects, particularly regarding safety. We here make generous assumptions that a thin (15 μm), low-density polymer separator can be used, although we note that a thicker material with better chemical resistance^16^ may be needed in reality. We additionally assume that each gram of case provides 6 mL of cell volume, around two to three times that which is achieved with 21700 cells,^17^ on the basis that the cell case can in part be structural in its application. This mass is then not additional to what is already required for the application. In addition, one can consider making cells larger than a 21700 and thus benefiting from a cube square law reduction in mass. Although, as we discuss later, if the cell is pressurized, one does not get this benefit. We take the aluminium and copper current collectors to be 1.5 μm thick, again pushing the limits of what is currently achieved.^18,19^ These are deliberately optimistic assessments, both to be lenient on the electrolyte requirements, as well as to hopefully account for improvements in battery manufacturing by the time lithium–air batteries become practical. We also add a 50% excess of the stoichiometric amount of lithium to account for loss to the SEI and other lithium sinks.^20,21^ We assume all separator and carbon pores are filled with electrolyte and that there is a slight, 10 μm thick, excess of electrolyte.

We consider our example cell [ESI-Excel file†] in Table 2, highlighting that due to the high porosity of carbons typical in Li–air batteries and our ultra-thin current collectors, the electrolyte contributes 40% of the mass of the battery. Since Li_2_O_2_ occupies a smaller volume than Li, mole for mole of Li, the “volume occupied” in the electrode by the electrolyte decreases as the lithium metal is stripped and Li^+^ is deposited as Li_2_O_2_ on the cathode. This results in the oxygen thickness having a negative value in Table 2. In practice, this could mean more of the cell would be occupied with gas, potentially aiding oxygen diffusion in cells where the electrolyte completely fills the pores of the cathode and separator. Previously, groups have reported that deliberately exacerbating this effect, by using approximately 20% less electrolyte than would completely fill the pores, results in better performance.^22^ We note, however, that this paper did not reach the deep discharges where the newly vacated space would become significant.

**Table 2:** Components in our example cell and their contributions to the cell's mass and volume. The mass fraction is compared to a cell at half discharge, and so the % mass values do not add up to 100%

Component	Mass (g) (fraction)	Thickness (μm)
Copper	1.2 (5%)	1.5
Lithium	2.3 (10%)	49
Separator	0.5 (2%)	15
Carbon	3.8 (17%)	100
Aluminium	0.4 (2%)	1.5
Electrolyte	9.6 (43%)	98
Casing	2.6 (16%)	3.8
Oxygen (full discharge)	3.6 (12%)	−7.7

**Fig. 1:**
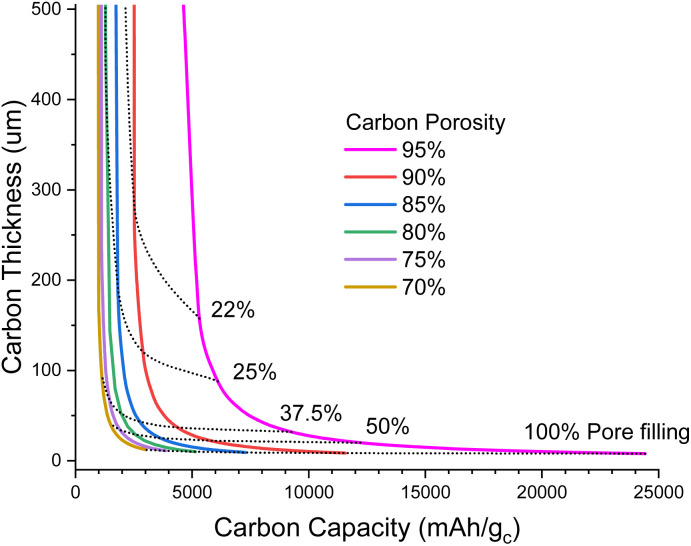
Plot of carbon electrode thickness as a function of carbon specific capacity to maintain 700 Wh kg^−1^ for a full cell (under the assumptions stated in the ESI-Excel file†) for various porosities. Dotted lines show the fraction of pores filled with Li_2_O_2_ at full discharge.

Fig. 1 illustrates the degree of pore filling that is required to maintain a cell energy density of 700 Wh kg^−1^ for the full cell. For example, for a highly porous carbon (95% porosity) that is 160 μm thick, around 22% of the pore volume must be filled with Li_2_O_2_ at the end of discharge, and this corresponds to around 5500 mA h g^−1^ carbon (mA h g_c_^−1^); note that this carbon capacity just corresponds to an amount of Li_2_O_2_ deposited in the pores per gram of carbon and is not an intercalation as in lithium-ion chemistry. In contrast, a carbon with 70% porosity of the same thickness would require more pore filling, around 34% to maintain 700 Wh kg^−1^, but this corresponds to a lower carbon capacity of 1000 mA h g_c_^−1^. The very steep rise of carbon thickness at low carbon capacities is due to the minimum degree to which we can shrink the inert cell parts, mostly electrolyte and casing, meaning that below a certain carbon capacity it is no longer possible to achieve 700 Wh kg^−1^, even with an infinitely thick carbon electrode.

When going to thinner carbon electrodes, the carbon capacity must increase to compensate for the greater inert mass, in turn increasing the fraction of the pores occupied by Li_2_O_2_, which eventually reaches unrealistic levels. In principle we can alleviate this by using more porous carbon, yet further shrinking the inert material mass and lithium excess, but our assumptions are already very optimistic. Thus, it seems unlikely that carbon thicknesses <50 μm will be practical (Fig. 1).

**Fig. 2:**
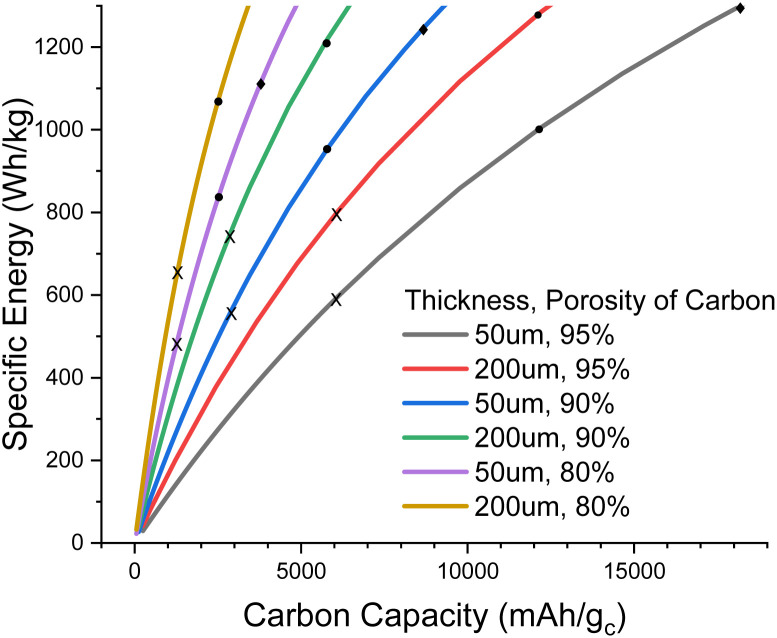
Plot of cell specific energy against carbon specific capacity for various carbon electrode thicknesses and porosities. X, ●, and ✦ mark 25%, 50%, and 75% of the pore being filled with Li_2_O_2_ at full discharge, respectively. 75% pore fill is only visible in the 50 μm thick carbon case.

In Fig. 2, we explore the pore filling and corresponding carbon capacity for various cell specific energies. For example, a *ca.* 500 Wh kg^−1^ cell could be constructed using a 50 μm thick 80% porosity carbon with around 25% pore filling, corresponding to 1200 mA h g_c_^−1^. This lower bound to the carbon and hence electrolyte thickness also places requirements on the electrolyte transport properties. Particularly important is oxygen diffusion, which we shall now explore.

### Oxygen transport

For the cell to successfully discharge it needs to always have dissolved oxygen throughout its cathode at an appreciable concentration. We approximate this requirement as needing the oxygen concentration to be >0 across the cathode assuming steady-state diffusion from a saturated O_2_ source; this being the surface of the electrolyte in contact with the overhead oxygen gas. While a cell could initially discharge at a higher rate, this would result in the oxygen-facing side becoming covered in discharge product, and the compounding factors of the increased diffusion distance and increased tortuosity of the outer layers starving the inner layers of oxygen and hence stopping the cell from discharging.

We consider an important parameter (DC), that is the product of the O_2_ diffusion constant and the saturation O_2_ concentration, as a metric with which to rank the solvents. A simple analysis (see ESI-Excel file†) leads to the conclusion that a practical solvent must have a DC greater than around 4.4 nmol m^−1^ s^−1^. This, however, is an excessively optimistic assessment as it assumes a bulk liquid unobstructed by pores. We can achieve more realistic values by accounting for the tortuosity and porosity of the carbon. With these effects taken into account [ESI-Excel file†], the DC rises to around 9.7 nmol m^−1^ s^−1^, approximately double the original estimate, but it is now closer to some other previous estimates.^23^ However, it is still notably lower than values observed elsewhere that were shown experimentally to be needed to achieve practical discharge rates.^24,25^ Possibly this is due to variation in electrode thickness, which is often greater than the 100 μm assumed here, or that excess electrolyte was used resulting in an increase to the diffusion distance. It may also be that the rate of Li_2_O_2_ formation is also a function of the dissolved O_2_ concentration in the electrolyte. This would greatly exacerbate the uneven deposition of Li_2_O_2_ in the cell and lead to our underestimate. Our tortuosity estimate may also be optimistic, given it assumes perfectly spherical particles, which is unlikely given the disordered nature of carbon and the Li_2_O_2_ depositions that often lead to bottlenecks that clog pores. We also note that the increase in capacity with increasing oxygen pressure *P*_O_2__ is only linear for low pressures and has a point of diminishing returns for very high rates.^25^

Based on the more pessimistic, but more practical, previous work, we can say that DC > 52 nmol m^−1^ s^−1^ is sufficient to enable the high (>1 mA cm^−2^) rate cycling^25,26^ that we require from our example cell (ESI†). It is likely, however, that with a thinner electrode and a minimalised amount of electrolyte, this requirement could be reduced significantly (up to 5 times).

### Pressurising the cell

We can relax the restrictions placed on the electrolyte by increasing the oxygen partial pressure. This, through Henry's law, increases the dissolved oxygen concentration at saturation. Already most cells run at a *P*_O_2__ far greater than atmospheric pressure, and gains in rate performance have been reported up to *P*_O_2__ of 20 bar, 100 times atmospheric pressure.^25^ We first consider using a pressurized oxygen tank to store the oxygen for the cell.

If we construct both the cell casing and pressure vessel with a high-performance material, say carbon fibre, even with a highly optimistic safety factor, 
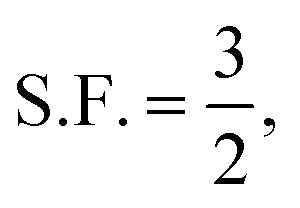
 and a spherical vessel, this results in a 10% increase in the mass of the cell. This is, possibly, an acceptable increase. However, taking a more realistic S.F. = 2 and *K* = 2 corresponding to an infinite cylinder and constructing only the O_2_ pressure vessel out of carbon fibre with the cell casing being constructed of high-strength steel, this increases to 27% (see the ESI-Excel file† for calculations). Thus to achieve our 700 Wh kg^−1^ target, the battery without the tank and additional O_2_ mass would need to have a specific energy density of 890 Wh kg^−1^. This is a very high target so, for our purposes, we consider it practical, and similar conclusions have been reached in previous reports.^4^ While in principle we could use some other store of oxygen, such as hydrogen peroxide, this departs from the intent of a lithium–air battery.

Alternatively, we could pressurize atmospheric air, for example, using a device like a turbocharger on a car, with a compressor for incoming air, and a turbine to extract the energy of the expanding, now oxygen-depleted, exhaust gas.

**Fig. 3:**
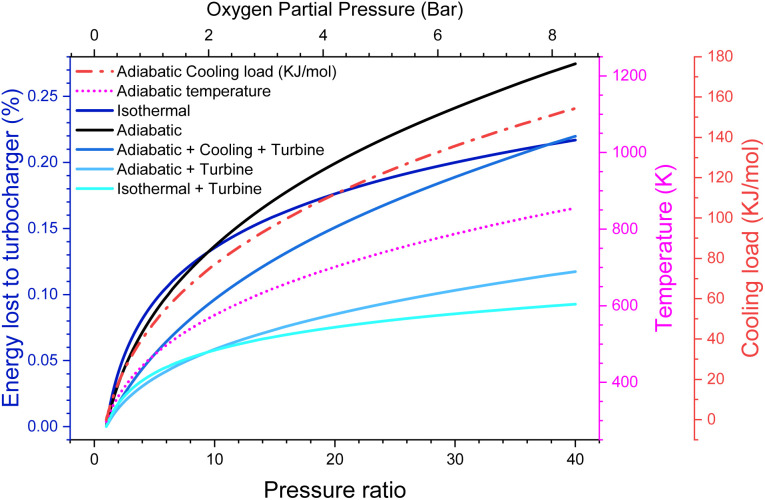
Efficiency of adiabatic, isothermal and adiabatic with cooling compression schemes. Additionally, the temperature after adiabatic compression and the thermal load for the isothermal and cooled cases are shown.

There are energy implications for this. While, in principle, adiabatic gas compression is reversible, real compressors typically only have an efficiency of around 80%.^27^ Even going to moderate pressures of 20 bar (*ca. P*_O_2__ = 4 bar), we lose 9% of the energy the battery generates just to run the compressor, even with a turbine to recover the outgoing energy. This makes our efficiency target challenging, although not impossible to reach and possibly more efficient turbines/compressors are available. However, of much more serious concern is that the gas entering the cell is now at 700 K. We could cool this gas, but that would lower the pressure and put a significant cooling load on the battery, equivalent to around 0.58 V of overpotential (see the ESI-Excel file†]. It also notably reduces the amount of work that could be extracted from the outgoing gas stream, causing around 15% of the energy to now be lost (Fig. 3).

Instead, we could intercool the compressor and put this heat into the turbine. This itself presents a significant engineering challenge. However, it does in theory give an option for the cell to remain at around room temperature and to still achieve high pressure with minimal efficiency loss. We leave it to the reader to decide which, if any, of these scenarios seems plausible. In any case, it seems unlikely that pressure ratios much greater than 20 (*i.e.*, *P*_O_2__ = 4 bar) could reasonably be achieved due to the energy losses.

Compounding this is the issue that the cell casing needs to be reinforced to take the additional pressure on the cell. It has been reported that 18650 batteries have a burst pressure of around 35 bar.^28^ This roughly agrees with theoretical calculations [ESI-Excel file†] showing that the mass of a steel cylinder able to hold 35 bar would make up around 16% of the total cell mass. By using higher-strength steels, the pressure rating could likely be extended, and one could imagine other materials, such as carbon fibre, allowing even greater pressures to be reached. Additionally, we do not include here the mass of a turbocharger required for this compression. We propose that using pressures significantly above 40 bar starts to become quite challenging from a weight and safety perspective.

This means that the electrolyte needs to have diffusion(O_2_) × Henry’s law constant of >10 nmol bar^−1^ m^−1^ s^−1^, if using *P*_O_2__ = 5 bar. This is approximately that of diglyme + 1 M LiTFSI,^26^ although, as noted before, this requirement can possibly be reduced by using a thinner electrode.

We note that our drone example operates at significantly lower air pressure, however, it also has a significantly lower rate requirement. Thus, it could operate at 1/3rd the pressure (equivalent to being *ca.* 10 km up) with the same boost requirements.

A drone operating at high altitudes experiences extremely dry air, but this is not the case at low altitudes.^29^ Since excess water can have a significant negative impact on battery performance,^30^ it should ideally be excluded from the cell, or its amount controlled. By pressurising the cell, water can, in principle, condense out. It's partial pressure at 298 K is 3.2 kPa, thus, with *P*_O_2__ = 500 kPa, it will take around 150 cycles before as much H_2_O has entered the cell as there is O_2_ on a single discharge. Alternatively, this means the relative humidity of 298 K air would be reduced to at most 5%, which has previously be reported to significantly extend cell life.^30,31^ In contrast, at these pressures, the second most significant contaminant CO_2_ will not undergo a phase change^32^ and must be separated some other way.

### Flow through the cell

We could bypass the oxygen diffusion issue by flowing electrolytes through the cell. It has previously been reported that pumping fluid through cells can lead to significant rate performance increases, similar to what is seen with pressure increases.^33^

We should, however, assess the energy requirements of pumping the fluid through a nanoparticle bed. To do this, we use the Darcy equation to calculate the pressure drop, along with the Kozeny–Carman equation to calculate the permeability of carbon particles. We use the assumption that the carbon particles are perfect spheres, with some porosity. We again make fairly generous assumptions that we are pumping through 100 nm spherical particles, of 55% porosity (corresponding to a fully discharged state), and a 200 μm electrode [ESI-Excel file†].

**Fig. 4:**
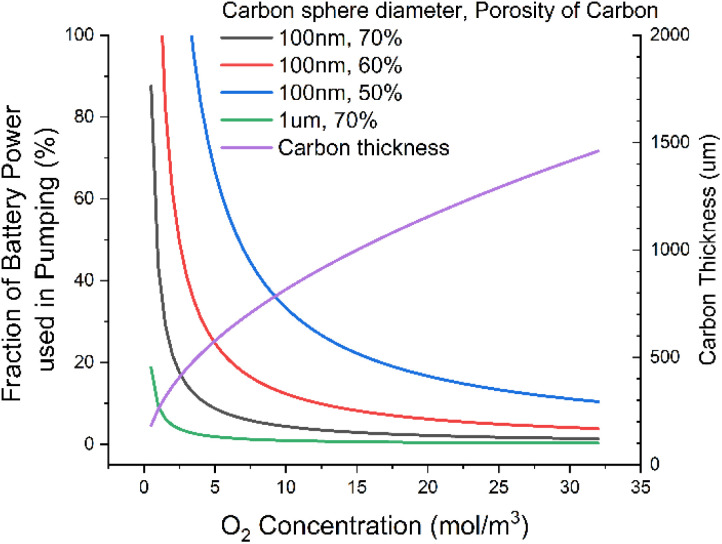
Fraction of power used to pump fluid through the cell as a function of O_2_ concentration for various carbon thickness and porosities. At lower O_2_ concentrations, the cell carbon thickness is made thinner to maintain the power per unit volume of fluid at a constant.

We find that relaxing requirements on the electrolyte, say by dropping DC to *ca.* 20 nmol m^−1^ s^−1^, results in >50% of the output energy of the cell being used just to power the pump (Fig. 4). This seems an unacceptable level of consumption for what is a modest relaxation in electrolyte requirements. We can significantly reduce this power consumption by using larger carbon particles, say 1 μm diameter, which would drop this power consumption to *ca.* 2%, a much more reasonable number. But this would require the discharge product particle sizes to be around a micron. These have previously been reported,^34^ but achieving them requires the Gibbs free energy of LiO_2_ solvation to be very favourable. This typically requires high acceptor numbers^35^ (AN) and donor numbers^36,37^ (DN). This in turn tends to lead to higher viscosity^1^ than we have here assumed; this then leads to a vicious cycle of higher viscosity leading to higher pumping power in turn needing to be offset by using larger diameter carbon particles and so needing larger discharge product enabled by higher AN/DNs.

It might be possible to avoid this by having an ionic region and a lower viscosity diffusion region, say with an ionic liquid and a diluent. This ultimately is the proposal we are led to if we do not consider pumping the fluid. Additionally, as we discuss later, the polar parts of the electrolyte molecule are also more likely to be attacked and lead to breakdown so likely should be minimised. But we note that pumping with large carbons could enable a higher viscosity regime if the right balance of viscosity, oxygen and discharge product could be reached.

It may also be possible to combine diffusion and pumped flow, say by having micron-sized agglomerations of high-surface-area nanoparticles surrounded by comparably large pores through which fluid can flow,^38^ thus creating a hierarchy of flow. This and other ideas for reducing pressure drop are explored far more in the field of redox flow batteries.^39^

### Evaporation

For the cell to have a reasonable lifetime, the electrolyte must evaporate sufficiently slowly. Although it may be possible to top the cell up at some maintenance interval, this should not be done too often.

**Fig. 5:**
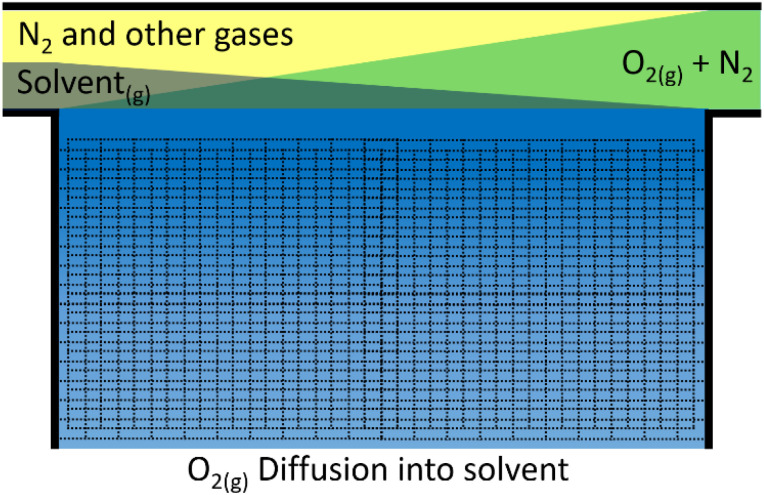
Schematic of assumed oxygen diffusion into the electrolyte of the cell from the pressurised air. Air enters the cell from the right and flows to the left. During this time the N_2_ and other gases concentration remains approximately the same in the gaseous phase (yellow), the O_2_ concentration (green) drops, while the solvent (grey) evaporates into the gas stream. The electrolyte (blue) at the gas interface is highly oxygenated (dark blue) and becomes less oxygenated further away from the interface (light blue).

To assess the rate of evaporation we again assume that at the interface of the gas/liquid boundary the two components are in equilibrium. This time, the liquid partial pressure in the gaseous phase is of interest, *i.e.* the concentration of the grey solvent vapour just above the blue electrolyte in Fig. 5. If we use the Clausius–Clapeyron equation and assume the vapour behaves as an ideal gas with much greater volume than the liquid phase, we get eqn (21), which relates vapour pressures between temperatures.

We now invoke Trouton's rule, which states that the entropy of vaporisation of a liquid at its boiling point is roughly the same for various liquids and equal to approximately 10.5*R*. This implies a linear relationship between the enthalpy of vaporization and boiling point.^40^ This rule can break down if the entropy change of vaporisation is far from the “ideal” case, such as if the liquid phase has extensive hydrogen bonding, though it is likely such molecules would be destroyed in the basic pH conditions anyway, or if the gas phase contains dimers, however the rule is applicable to a variety of solvents.

Taking these assumptions provides us with a convenient link between the more intuitive boiling point and the vapour pressure at our temperature of interest.

We must now assess what the upper limits of acceptable vapour pressure are. If we take a cell operating at ambient pressure and discharging at 60% O_2_, we find that the vapour pressure must be <120 Pa for the cell to last 100 cycles and lose only 20% of its electrolyte [ESI-Excel file†]. Significant gains can be made by upping the pressure in the cell. At 20 bar, the acceptable vapour pressure increases to 2400 Pa due to the oxygen partial pressure now being much greater relative to the solvent partial pressure. This would lead to boiling points needing to be greater than 220 °C and 130 °C, respectively, or alternatively, enthalpies of vaporisation of 43 kJ mol^−1^ and 35 kJ mol^−1^.

Because of the logarithmic relationship between the partial pressure and the boiling point of the material, changes in the requirements on vapour pressure have a relatively small impact on the boiling point requirements. For example, halving the acceptable vapour pressure, say by requiring 200 cycles or accepting only 10% electrolyte loss, only leads to a rise in the required boiling point of 20 °C.

We note here that adding salt can reduce the vapour pressure and hence the boiling point of a pure solvent in an electrolyte. Thus, it is necessary to consider the combined system, including salt and any other dissolved species and interactions with solids, not just the pure solvent contribution.

One could also significantly ease the lower limit of the boiling point by placing a suitable, oxygen-selective but solvent-repelling membrane onto the exhaust. This could be a simpler alternative to pressurising the overhead gas, albeit without the advantage of improved oxygen diffusion. The design of such a membrane is, however, beyond the scope of this article and we do not consider the case of a cell that has one.

As we discuss later, the diffusion of oxygen, and other species, also scales inversely with viscosity and, as we have related viscosity to the boiling point, this ends up also putting an upper bound on the boiling point of around 300 °C, corresponding to a viscosity around 5 mPa s.

Still, we also highlight that low vapour pressure is desirable as this reduces the flammability of the solvent,^41^ as well as reducing the risk of any exhaust being toxic or contributing heavily to pollution or global warming potentials, but conclude that an acceptable lower bound on the boiling point is around 160 °C.

### Oxygen solubility in the electrolyte

We now turn our attention to what sort of solvent would best dissolve oxygen. For this we shall use the Hansen solubility parameters. These are selected as they break a solvent down into dispersion (*δ*_D_), polar (*δ*_P_) and H-bonding (*δ*_H_) parameters. They also have the decided advantage of being related, *via* the cohesive energy (*E*), to the enthalpy of vaporisation (*H*_v_), which we have already put bounds on. They of course do not capture all possible bonding interactions, but they can at least provide a semi-quantitative description of the bonding interactions that a solvent needs to have.

The solubility of O_2_ in a particular solvent can be predicted by assessing the distance in the Hansen parameter space between the Hansen solubility parameter (*R*_H_) of the solvent and that of oxygen. It should be noted that, to a reasonable approximation, Hansen solubility parameters are independent of molecular size, so long as they have similar solvent structures, as the Hansen parameters are scaled by molar volume. For example, all members of the alkane and perfluorocarbon series have similar Hansen parameters to other members in the series. This is useful as it implies that once we find a functional group with suitable O_2_ solvation characteristics, we can simply scale the chain length (and hence molar volume) to get the enthalpy of vaporization and boiling point we desire.

Considering oxygen has the parameters *δ*_D_ = 6.7, *δ*_P_ = 0, and *δ*_H_ = 3.8,^12^ we see the ideal solvent is one with low dispersion interactions and some hydrogen-bonding ability. However, given that hydrogen-bonding groups are likely to be deprotonated – and the presence of protons will cause other issues, *e.g.*, H_2_ evolution at the anode – low dispersion and some polarity are likely the most desirable properties. The increased polarity is to dissolve lithium salts. Functional groups that are closest to these requirements are, in order of appearance: perfluoroalkyls (PFC), siloxanes, CFCs, HCFCs, alkanes, alkenes, fluorinated ethers, long alkyl chain esters, ethers, amines, and ketoximes [ESI-Excel file†]. Reassuringly (and unfortunately), all of these functional groups have either already been considered for lithium–air batteries (perfluorocarbons, alkanes, ethers, amines), are ozone-depleting (CFC, HCFC), or have very likely degradation pathways (alkenes, esters, ketoximes). The possible exception is siloxanes, although they do degrade in the presence of strong bases (such as NaOH) and do not bind metal ions as strongly as ethers so, while worthy of investigation, may not be practical.

We note that by far the best functional group for oxygen solvation is the perfluoroalkyl carbon chain.^11^ This is due to fluorine's high electronegativity leading to weak dispersion interactions, as well as its larger size compared to hydrogen, increasing chain rigidity and resulting in weak intermolecular forces, and so oxygen is easily able to dissolve into the solution.^42^ PFCs also present excellent chemical resistance and so are a tempting choice for an oxygen-solvating group. Alkanes are another option, although more susceptible to attack.

As well as oxygen solubility, we also need to dissolve the lithium salt. For this, we desire a high-polarity solvent. This would likely reduce the oxygen-solvating ability, and to get around this we could use an amphiphile, *i.e.*, a molecule with both a polar and apolar end. One could also consider ionic liquids, which often have two sets of Hansen solubility parameters^43,44^ to capture their ability to dissolve both polar and non-polar molecules. This can be rationalised as them having an ionic component, due to the charge centres, connected to a less ionic component. Alternatively, this could be achieved by a polar molecule in some dilutant, as is often used in high-concentration locally concentrated solvents.

We may also desire to use the solvent to exclude H_2_O and CO_2_ from the cell. Considering the H_2_O and CO_2_ Hansen solubility parameters, we see already that by limiting dispersion, polarity and H-bonding, we are already minimising the CO_2_ and H_2_O solubility. Thus, within the constraints of our discussion, we are already maximising the solvent’s ability to exclude H_2_O in particular, but also CO_2_. Perfluorocarbons, for example, are hydrophobic and much worse at dissolving CO_2_ than O_2_. Having said that, for solvents to be able to dissolve salts it is often necessary to increase the polarity and H-bonding to the point that H_2_O and CO_2_ dissolve well. Thus with current solvents, H_2_O and CO_2_ cannot be excluded (*i.e.*, they are often highly soluble) as has been noted by others.^26^ This is particularly so in the presence of lithium salts.

### Diffusion and viscosity

The final part of the oxygen transport that we can optimise is oxygen diffusion, which is also strongly related to ionic transport *via* the solvent's viscosity. We can use the Stokes–Einstein equation to estimate the diffusion of oxygen through our solvents. The Walden plot relates the log(ionic conductivity) of an electrolyte to the log(1/viscosity). A line is drawn through the values corresponding to 0.01 M KCl_(aq)_ which is considered the ideal ionic conductor. Solvents falling above this line are considered “super-ionic” conductors, those below but near the line are good ionic conductors, and those far below the line are poor ionic conductors. It should be noted this is at best a semi-quantitative description,^45^ but it provides a basis for discussion.

Clearly, in both cases, a low-viscosity solvent would be desirable for the best transport. However, low viscosity generally results from weak molecular interactions, which generally manifest in a low boiling point, which runs counter to our previous discussion on evaporation. Unfortunately, the link between viscosity and boiling point is only approximately derivable^13^ and errors of around 30% can be expected when using it. However, given that we are interested in the logarithm of viscosity, this is not too concerning to us.

**Fig. 6:**
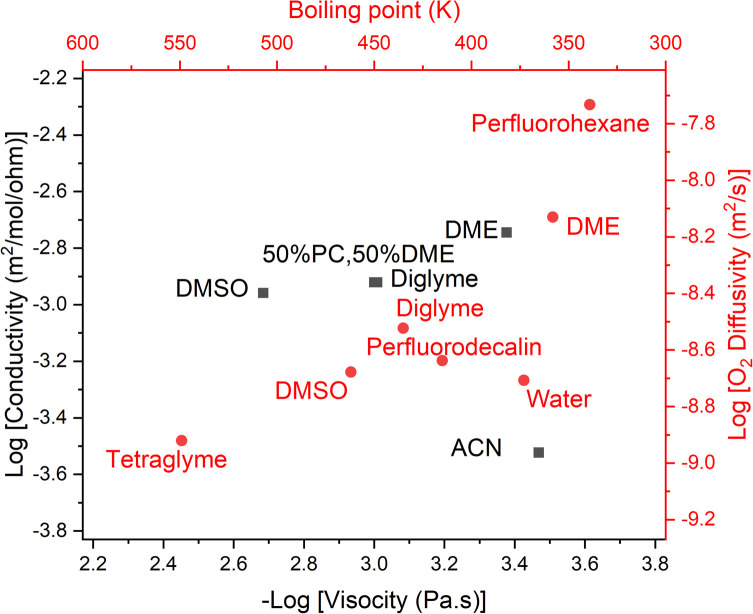
Walden plot (conductivity *vs.* viscosity, in black) also showing the approximate boiling point corresponding to a given viscosity, as well as the oxygen diffusivity corresponding to a given conductivity (in red). Common solvents are plotted and red vertical lines show the region that is considered acceptable for metal–air battery operation. The diagonal line represents the ideal conductivity for 0.01 M KCl in water. Citations for the data can be found in the ESI†. If our assumptions regarding the relationship between boiling point and viscosity and O_2_ diffusivity and conductivity were perfectly correct, the red and black dots for a given solvent would perfectly overlap. We reassuringly see close matches in most cases.

Using this we can create a modified Walden plot (Fig. 6) to plot both boiling point and viscosity on the *x*-axis and ionic conductivity and oxygen transport on the *y*-axis. We also add lines at a boiling point of 130 °C and 300 °C corresponding to around 5 mPa s of viscosity in the latter case.

It should be noted that the viscosity varies noticeably as a function of the solvents’ molar concentration (*i.e.* 1/molar volume), thus there can be significant variation in viscosity, even at the same boiling point. We here assume a molar concentration of the solvent of 8 M.

We consider an upper bound on the acceptable diffusion of a solvent to be around 5 mPa s. This arises as even using the solvents with the highest Henry's law coefficient, perfluorocarbons at around 20 mM bar^−1^,^46^ and a high oxygen partial pressure, *P*_O_2__ = 5 bar and *r*_O_2__ = 1.45 Å, which is regressed from the data [ESI†], one achieves a DC ≈ 60 nmol m^−1^ s^−1^ with a viscosity of 5 mPa s at the limit of what we have previously considered acceptable.

Given that virtually all solvents have worse oxygen solubility characteristics than perfluorocarbons, a practical cell would need to be on the lower end of the acceptable viscosity range, and hence also have a lower boiling point. We can also consider that most electrolytes have very satisfactory ionic conductivity and thus diluting the typical electrolytes with a less polar additive is likely to be an attractive route to better battery performance.

### Ionic transport

As previously alluded to, the cell must have sufficient Li^+^ ion conductivity, both to support even Li plating on the anode as well as to enable Li_2_O_2_ precipitation. In theory these can both be modelled by the Sand's^47^ time, albeit with the simplification that the carbon surface is considered a single plane. The situation is more complicated than this, particularly at the lithium anode due to the effects of the SEI and its inhomogeneity,^48^ however, we can still use it to place a lower bound on the required conductivity.

In principle, we desire to stay reasonably far away from the Sand’s time to avoid Li dendrite growth or depletion of Li^+^ from the carbon cathode. However, we find that for the low transport distances (*ca.* 100 μm) and large capacities that we are considering, it is easier to enter a regime below the critical current where the Sand’s time does not apply, *i.e.*, the steady-state concentration at the lithium-poor electrode is non-zero.

We may still want to stay at, say, half this value to ensure there is still appreciable Li^+^ throughout the cell. If we take fairly viscous 5 mPa s electrolyte, even assuming very large solvation shells making the Li^+^-ion effective radius, *r*_eff_, 1.5 nm wide, even a 200 mM Li^+^ concentration would result in a limiting current density of 2 mA cm^−2^. Even a much more poorly solvating electrolyte with a transference number of 0.05 could work with an effective radius of 1 nm. We note that we are working with the Nernst–Einstein approximation of non-interacting particles. With interacting particles, the transference numbers may become a lot less favourable.^49^ But, so long as there is a reasonable degree of solvation of the lithium salt, relatively low concentrations of Li^+^ are possible.

### Discharge product solubility

Closely related to ionic transport for conductivity is the issue of dissolving the discharge product. It has been widely discussed that better dissolution of Li^+^ and O_2_^−^ leads to larger crystals of discharge product due to the solution mechanism of Li_2_O_2_ formation being favoured.^50^ The Gibbs free energy of dissolution has been closely tied to the acceptor number and donor number of the solvent system in question.^36^

As we are yet to put bounds on the AN and DN, these could be considered to be free parameters that in principle we could maximise. However, as has been noted previously, materials with both high acceptor and donor numbers lead to strong intermolecular forces leading to high viscosities, running counter to our previous discussion.^1^

We thus first turn our attention to what mA h g_c_^−1^ capacity can be achieved without using a solution mechanism for Li_2_O_2_ growth. The surface mechanism leads to a thin coating on the order of 6 nm thick:^50^ we find that with a relatively high-surface-area carbon, say Ketjenblack,^51^ this corresponds in theory to >5000 mA h g_c_^−1^, far in excess of what we are requiring of our practical cell [ESI-Excel file†]. This is also born out experimentally with ultra-high-surface-area porous graphene providing 15 000 mA h g_c_^−1^ capacities in a triglyme (low DN and AN)-based electrolyte.^52^

Using such high surface area carbons does increase the risk of breakdown-product build-up: since some degradation mechanisms involve carbon oxidation at the carbon–Li_2_O_2_ interface, the much higher surface area of Li_2_O_2_ in contact with the electrolyte will likely lead to more degradation.^2^ While the authors are not aware of a paper that directly relates surface area to an increased rate of breakdown, it certainly seems a likely outcome. However, with the aim of minimising the electrolyte requirements, we shall put aside this concern and assume it can be dealt with in some other way, say by treating the carbon surface to make it hydrophobic^53^ and making the electrolyte suitably resistant to breakdown.

## Discussion

In the Results section, we discussed the cell geometry, transport properties, evaporation, and ways that a realistic energy density of a lithium–air cell can be achieved with the lowest constraints or restrictions on each of these components. All of these system and component properties are inevitably coupled, and while all considered criteria can be achieved by some existing solvent, and, with some judicious design all simultaneously, we are yet to identify a workable solvent that is resistant to breakdown, even for non-practical cells. Thus, we now turn finally to the most challenging criterion: that of electrolyte degradation. We thus discuss the causes of electrolyte degradation, suggest ways to avoid this and present possible options for solvents to fulfil these requirements. We present this analysis now in the discussion section since it departs from the more mathematical previous sections due to the difficultly of describing degradation with simple mathematical formulae, without resorting to highly simplified models.

### Electrolyte degradation

Mechanisms commonly proposed for electrolyte breakdown are H and H^+^ removal, possibly *via* elimination as well as nucleophilic attack, electrochemical oxidation or reduction,^54^ or reaction with radicals leading to oxidation.^2^ It is currently thought that the major cause of degradation in glyme-based solvents is attack by singlet oxygen, with 7% of all oxygen being used by the cell turning to singlet oxygen and up to 75% of all degradation products coming from singlet oxygen attack.^55,56^

To decrease the effect of singlet oxygen we could quench (converting it to its triplet state) it quicker, stop its formation or slow its degradation reaction with our solvent. When quenching the reaction it is necessary to use a physical quencher, as the amount of singlet oxygen produced is so much that any chemical quencher would be consumed too rapidly.^56^

In proton-containing species, singlet oxygen lifetime is on the order of 10–100 μs.^57^ This lifetime is not quite the same as the rate of chemical reaction as it also includes quenching reactions, however, it does indicate the speed at which we need to quench the singlet oxygen to prevent it from reacting. Fully fluorinated solvents have singlet oxygen lifetimes around 1000× times this^58^ and comparable to the lifetime of singlet oxygen in free gas. This shows fluorinated chains have a remarkable resistance to singlet oxygen and it is likely that a purely fluorinated chain would be resistant to singlet oxygen attack. However, fully fluorinated chains have very little polarity and thus it is a struggle to dissolve the 200 mM of Li salt to achieve acceptable Li-ion conductivity, as discussed previously.

Preventing any singlet oxygen formation presents a significant challenge, as it can be formed from superoxide disproportionation even on discharge and at a modest charge voltage of 3.55 V.^59^ In principle, one could avoid this by using a redox mediator on both discharge and charge to make the formation of singlet oxygen energetically unfavourable.

It thus seems necessary to quench down the singlet oxygen, at least to some degree. If this was used as the sole means of preventing singlet oxygen formation, it would require quenching on timescales of <1 μs. This does appear achievable using existing quenchers,^4^ but these quenchers in turn cause issues, as they are often electron-rich to enable intersystem crossing, and typically contain nitrogen atoms with lone pairs. These quenchers can then be attacked by the lithium metal,^60^ by other reactive oxygen species or even *via* electrochemical attack.^55^ While steps have been made in addressing these issues, an effective long-term mediator has yet to be developed.

Even with a suitable quencher, around 25% of the degradation is unaccounted for. Early works suggested that, for a hydrophobic carbon, although there is an initial breakdown of the carbon, this is not the major contribution after the first cycle.^53^ This suggested that the breakdown is caused by a reaction of either the superoxide or the peroxide.

Previous works have discussed using H-bond dissociation energies and p*K*_a_ (*i.e.*, deprotonation energies) as measures for H and H^+^ removal, respectively. Given the non-aqueous conditions that lithium–air batteries operate in, it is not immediately obvious what should be considered an acceptable p*K*_a_ or hydrogen-bond dissociation energy.

Such discussion is also straying beyond that which engineering adjustments of the battery can clearly address, given that Li_2_O_2_ production is intrinsic to the batteries' operation and is thus unavoidable.

Still, given the more recent understanding of the importance of singlet oxygen in causing degradation and the steady development of methods to quench it, a better understanding of the mechanism of this remaining degradation may be beneficial for preventing the remaining degradation observed in LABs.

### Future electrolytes

**Fig. 7:**
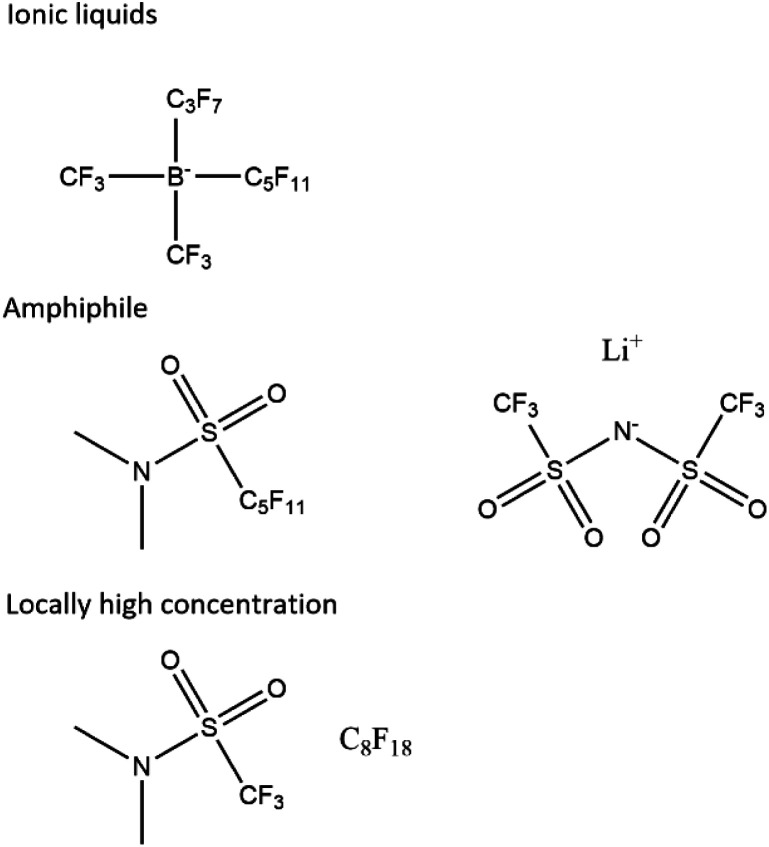
Proposed electrolyte systems. Other ionic liquids exist, and the sulphonamide is just an example ionic functional group that has been proposed before.^61^

We propose that future electrolytes will likely have a non-polar region for oxygen diffusivity and a highly polar region for the solvation of lithium ions and low viscosity. This could be achieved by, for example, a locally high-concentration electrolyte, a single amphiphile molecule or an ionic liquid, possibly with a dilutant (Fig. 7).

A non-polar region with suitable properties is likely to be a perfluorinated or hydrocarbon chain. The challenge then lies in creating a suitable polar region and critically one that is resistant to chemical attack. While proposing an exact structure lies beyond the scope of this work, we note that encouraging functional groups have been sulphone^61^ and amides,^55^ particularly with the latter's potential to quench singlet oxygen. Ionic liquids also present interest avenues with quaternary ammonium ions^62^ and imidazole^63^ being suggested as promising by previous work.

We also propose that the lithium perfluoroalkyl borates may be an interesting class of compounds to investigate further. They are the charge-reversed versions of the perfluorinated ammonium chlorides, which are ionic liquids.^64–66^ The strong electron-withdrawing effect of the perfluorinated chains around the boron strengthens the B–C bond and similar electrolytes have been used in battery systems.^67,68^ Additionally, in the undiluted form, they would enable perfect lithium transference. Unfortunately, they are not trivial to synthesise^69,70^ and our own efforts to make them have been unsuccessful.

## Summary and conclusions

By analysing the transport of O_2_ and Li^+^ ions, as well as the cell geometry required to achieve a 700 Wh kg^−1^ cell level LAB, we have placed requirements on the properties that a practical electrolyte must have. Then, using pressurisation and an optimised geometry we have relaxed these requirements. We also considered the effect of flow and using a pressurized source of pure O_2_ instead of atmospheric air. We summarise our proposed relaxed requirements for the electrolyte in Table 3.

**Table 3:** Proposed requirements on future electrolytes. The limits given below are those that result from other constraints on the electrolyte, as outlined in the second column (rationale)

Limit	Rationale
130 °C < *T*_boil_ < (300 °C)	Temperature constrained by evaporation
Viscosity < 5 mPa s	Viscosity limited by O_2_ transport
5 mM bar^−1^ < H_O_2__	O_2_ transport limited
Density < 2000 kg m^−3^	Needed to achieve 700 Wh kg^−1^

In Table 4 we present the battery design guidelines we used to create these relaxed requirements and other ways to relax these requirements that were considered but not used in our cell. We make available an Excel spreadsheet as ESI† with all the calculations used in this paper so that others can explore further.

**Table 4:** Proposed guidelines for battery design

Guideline	Rationale
*P*_O_2__ < 5 bar	High pressurization costs or consumes too much energy. It also adds a significant cooling load
	Closed system adds too much weight
mA h g_c_^−1^ > 2000	Required to achieve >700 Wh kg^−1^
Carbon porosity >80%	Otherwise pore filling becomes unrealistic
50 μm < Carbon thickness < (200 μm)	Lower limit to achieve 700 Wh kg^−1^
	Upper limit constrained by O_2_ diffusion
Flow only practical if carbon pores > ≈500 nm	Constrained by power to pump fluid

Though it is challenging to fulfil all the requirements placed on the electrolyte simultaneously, we find that by using a thin (50–200 μm), highly porous carbon, pressurising the incoming atmospheric air (*P*_O_2__ = 5 bar), and targeting a molecule with a perfluoroalkyl chain, the length of which is adjusted such that the solvent boiling point is around 200 °C, we are likely to be able to create an electrolyte to satisfy the requirements for a practical LAB. However, this choice of electrolyte is unlikely to meet the criterion for sufficient lithium-ion transport. This is due to there being no known polar functional group suitably resistant to attack in the Li–air system. Thus, we are currently unaware of a solvent that will dissolve a lithium salt without the electrolyte also being liable to decomposition. Thus, a critical goal towards a practical lithium–air electrolyte is to find a solvent with an associated functional group that has both sufficient chemical stability to operate in the cell, while also sufficiently solvating lithium ions to allow a high enough concentration of dissolved ions and an acceptable level of Li^+^-ion transport.

## Conflicts of interest

CPG is a cofounder and a shareholder in a fast-charging battery company (Nyobolt).

## Supplementary Material

FD-248-D3FD00091E-s001

FD-248-D3FD00091E-s002

## References

[cit1] Husch T., Korth M. (2015). Phys. Chem. Chem. Phys..

[cit2] Feng S., Chen M., Giordano L., Huang M., Zhang W., Amanchukwu C. V., Anandakathir R., Shao-Horn Y., Johnson J. A. (2017). J. Mater. Chem. A.

[cit3] Liu T., Vivek J. P., Zhao E. W., Lei J., Garcia-Araez N., Grey C. P. (2020). Chem. Rev..

[cit4] Gallagher K. G., Goebel S., Greszler T., Mathias M., Oelerich W., Eroglu D., Srinivasan V. (2014). Energy Environ. Sci..

[cit5] Matsuda S., Ono M., Yamaguchi S., Uosaki K. (2022). Mater. Horiz..

[cit6] Barrande M., Bouchet R., Denoyel R. (2007). Anal. Chem..

[cit7] Xu W., Zhang K., Zhang Y., Jiang J. (2022). Water Resour. Res..

[cit8] Ibrahim A., Ryu Y., Saidpour M. (2015). Mod. Mech. Eng..

[cit9] Echtermeyer A. T., Lasn K. (2014). Int. J. Hydrogen Energy.

[cit10] Elliott R. W., Watts H. (1972). Can. J. Chem..

[cit11] Díaz de los Ríos M., Hernández Ramos E. (2020). SN Appl. Sci..

[cit12] Sato T., Hamada Y., Sumikawa M., Araki S., Yamamoto H. (2014). Ind. Eng. Chem. Res..

[cit13] Bird R. B. , Stewart W. E. and Lightfoot E. N., Transport Phenomena, Wiley, New York, Revised edn, 2007

[cit14] McCloskey B. D., Scheffler R., Speidel A., Girishkumar G., Luntz A. C. (2012). J. Phys. Chem. C.

[cit15] Zhang W., Shen Y., Sun D., Huang Z., Zhou J., Yan H., Huang Y. (2016). Nano Energy.

[cit16] Nasybulin E., Xu W., Engelhard M. H., Nie Z., Li X. S., Zhang J.-G. (2013). J. Power Sources.

[cit17] Waldmann T., Scurtu R.-G., Richter K., Wohlfahrt-Mehrens M. (2020). J. Power Sources.

[cit18] Chu H.-C., Tuan H.-Y. (2017). J. Power Sources.

[cit19] Yang L., Weng W., Zhu H., Chi X., Tan W., Wang Z., Zhong S. (2023). Mater. Today Commun..

[cit20] Louli A. J., Coon M., Genovese M., deGooyer J., Eldesoky A., Dahn J. R. (2021). J. Electrochem. Soc..

[cit21] Gunnarsdóttir A. B., Amanchukwu C. V., Menkin S., Grey C. P. (2020). J. Am. Chem. Soc..

[cit22] Matsuda S., Yamaguchi S., Yasukawa E., Asahina H., Kakuta H., Otani H., Kimura S., Kameda T., Takayanagi Y., Tajika A., Kubo Y., Uosaki K. (2021). ACS Appl. Energy Mater..

[cit23] Torayev A. , PhD thesis, Université de Picardie Jules Verne, 2019

[cit24] Read J., Mutolo K., Ervin M., Behl W., Wolfenstine J., Driedger A., Foster D. (2003). J. Electrochem. Soc..

[cit25] Nemanick E. J., Hickey R. P. (2014). J. Power Sources.

[cit26] Gittleson F. S., Jones R. E., Ward D. K., Foster M. E. (2017). Energy Environ. Sci..

[cit27] Kusztelan A., Yao Y. F., Marchant D. R., Wang Y. (2010). Int. J. of Thermal & Environmental Engineering.

[cit28] Coman P. T., Rayman S., White R. E. (2016). J. Power Sources.

[cit29] Colombo P., Fassò A. (2022). Meas. Sci. Technol..

[cit30] Wang L., Pan J., Zhang Y., Cheng X., Liu L., Peng H. (2018). Adv. Mater..

[cit31] Guo Z., Li C., Liu J., Wang Y., Xia Y. (2017). Angew. Chem., Int. Ed..

[cit32] Eldevik F., Graver B., Torbergsen L. E., Saugerud O. T. (2009). Energy Procedia.

[cit33] Chen X. J., Shellikeri A., Wu Q., Zheng J. P., Hendrickson M., Plichta E. J. (2013). J. Electrochem. Soc..

[cit34] Liu T., Leskes M., Yu W., Moore A. J., Zhou L., Bayley P. M., Kim G., Grey C. P. (2015). Science.

[cit35] Kwabi D. G., Bryantsev V. S., Batcho T. P., Itkis D. M., Thompson C. V., Shao-Horn Y. (2016). Angew. Chem., Int. Ed..

[cit36] Johnson L., Li C., Liu Z., Chen Y., Freunberger S. A., Ashok P. C., Praveen B. B., Dholakia K., Tarascon J.-M., Bruce P. G. (2014). Nat. Chem..

[cit37] Burke C. M., Pande V., Khetan A., Viswanathan V., McCloskey B. D. (2015). Proc. Natl. Acad. Sci. U. S. A..

[cit38] Wang F., Xu Y.-H., Luo Z.-K., Pang Y., Wu Q.-X., Liang C.-S., Chen J., Liu D., Zhang X. (2014). J. Power Sources.

[cit39] Forner-Cuenca A., Brushett F. R. (2019). Curr. Opin. Electrochem..

[cit40] McLachlan D., Marcus R. J. (1957). J. Chem. Educ..

[cit41] Hess S., Wohlfahrt-Mehrens M., Wachtler M. (2015). J. Electrochem. Soc..

[cit42] Dias A. M. A., Freire M., Coutinho J. A. P., Marrucho I. M. (2004). Fluid Phase Equilib..

[cit43] Agata Y., Yamamoto H. (2018). Chem. Phys..

[cit44] Kobayashi Y., Tokishita S., Yamamoto H. (2020). Ind. Eng. Chem. Res..

[cit45] Harris K. R. (2019). J. Phys. Chem. B.

[cit46] Schürmann A., Haas R., Murat M., Kuritz N., Balaish M., Ein-Eli Y., Janek J., Natan A., Schröder D. (2018). J. Electrochem. Soc..

[cit47] Bai P., Li J., Brushett F. R., Bazant M. Z. (2016). Energy Environ. Sci..

[cit48] Wasalathanthri R. N., Akolkar R. (2022). J. Electrochem. Soc..

[cit49] Fong K. D., Self J., McCloskey B. D., Persson K. A. (2020). Macromolecules.

[cit50] Aurbach D., McCloskey B. D., Nazar L. F., Bruce P. G. (2016). Nat. Energy.

[cit51] Tashima D., Yoshitama H., Otsubo M., Maeno S., Nagasawa Y. (2011). Electrochim. Acta.

[cit52] Xiao J., Mei D., Li X., Xu W., Wang D., Graff G. L., Bennett W. D., Nie Z., Saraf L. V., Aksay I. A., Liu J., Zhang J. (2011). Nano Lett..

[cit53] Ottakam Thotiyl M. M., Freunberger S. A., Peng Z., Bruce P. G. (2013). J. Am. Chem. Soc..

[cit54] Lai J., Xing Y., Chen N., Li L., Wu F., Chen R. (2020). Angew. Chem., Int. Ed..

[cit55] Petit Y. K., Leypold C., Mahne N., Mourad E., Schafzahl L., Slugovc C., Borisov S. M., Freunberger S. A. (2019). Angew. Chem., Int. Ed..

[cit56] Mahne N., Schafzahl B., Leypold C., Leypold M., Grumm S., Leitgeb A., Strohmeier G. A., Wilkening M., Fontaine O., Kramer D., Slugovc C., Borisov S. M., Freunberger S. A. (2017). Nat. Energy.

[cit57] Bregnhøj M., Westberg M., Jensen F., Ogilby P. R. (2016). Phys. Chem. Chem. Phys..

[cit58] Day R. A., Sletten E. M. (2021). Curr. Opin. Colloid Interface Sci..

[cit59] Wandt J., Jakes P., Granwehr J., Gasteiger H. A., Eichel R.-A. (2016). Angew. Chem..

[cit60] Laino T., Curioni A. (2013). New J. Phys..

[cit61] Feng S., Huang M., Lamb J. R., Zhang W., Tatara R., Zhang Y., Guang Zhu Y., Perkinson C. F., Johnson J. A., Shao-Horn Y. (2019). Chem.

[cit62] Temprano I., Liu T., Petrucco E., Ellison J. H. J., Kim G., Jónsson E., Grey C. P. (2020). Joule.

[cit63] Tkacheva A., Zhang J., Sun B., Zhou D., Wang G., McDonagh A. M. (2020). J. Phys. Chem. C.

[cit64] Gupta O. D., Armstrong P. D., Shreeve J. M. (2003). Tetrahedron Lett..

[cit65] Alhanash H. B. A. , 2012

[cit66] Eike D. M., Brennecke J. F., Maginn E. J. (2003). Green Chem..

[cit67] Krossing I., Raabe I. (2004). Angew. Chem., Int. Ed..

[cit68] Zhou Z.-B., Takeda M., Ue M. (2003). J. Fluorine Chem..

[cit69] Adonin N. Y., Bardin V. V., Flörke U., Fron H. J. (2011). Russ. J. Gen. Chem..

[cit70] Bernhardt E., Henkel G., Willner H., Pawelke G., Bürger H. (2001). Chem. – Eur. J..

