# Supplementary material for: Engineering considerations for practical lithium–air electrolytes
Source: Faraday Discuss. 2023 Aug 1;248:355–80. doi: 10.1039/d3fd00091e (PMC10823492; doi:10.1039/d3fd00091e)
Supplement: FD-248-D3FD00091E-s001 [file FD-248-D3FD00091E-s001.pdf]

Electronic Supplementary Material (ESI) for Faraday Discussions.
This journal is © The Royal Society of Chemistry 2023

Electronic Supplementary Information

Engineering Considerations for Practical Lithium-air Electrolytes

James H. J. Ellison,[a] and Clare P. Grey*,[a]

[a, a] *Yusuf Hamied Department of Chemistry, University of Cambridge, Cambridge, United Kingdom*
* Corresponding Author

Table of requirements

In all categories higher performance is desirable. For specific energy and energy density the value of 700Wh/kg is chosen to compete with lithium metal batteries, such as those being developed by battery 500[1], which are close to achieving 500Wh/kg performance. Given that lithium air batteries are far from commercialisation we consider it likely that such batteries will have been developed by the time Li-air batteries themselves are viable. We also note we consider this energy density at the cell level.

For energy efficiency 80% is selected for the grid due to economic pressure of its entire value being derived from its ability to return a good faction of the energy put into it. It needs to compete with pumped hydro which achieves 80+% efficiency[2]. Additionally beyond a certain point it is cheaper to install additional overcapacity rather than store the energy[3]. For the car case competition comes from H_2 and other synthetic fuel storage, which struggle to achieve >70% efficiency[4]. Having said this, current cars only achieve ca. 30% efficiency[5] and so this criterion could be relaxed. For the drone case the cost of additional energy is unlikely to be significant. However, 60% is chosen on the basis that voltage widows above 5V become very challenging on charge, and high overpotentials on discharge affect energy density.

The lifetime for a solar drone was selected as 12 cycles, as this gives a total energy output of 8.4KWh/kg, surpassing that which could be achieved using hydrocarbon fuels. For the car 600 cycles to 80% was selected as for an EV with a range of 1,000Km this would give >480,000Km lifetime beyond that which most cars would reach before retirement[6]. For a grid battery, this would be cycled every day or more, and so ideally would last for many decades, 3,000 was selected only as an example and could be double to triple this for an ideal battery.

Grid lulls and swells can be as short as 4 hours[2,3]. Weather variations tend to be longer, on days up to week scales, even 6 months for seasonal variations. However, as these are less stringent requirements, we neglect them. A car with a modest 500Km range travelling at reasonable 120Km/hr speeds could discharge its battery in around 4 hours. The charge ideally would be even faster, possibly sub 1hr as is achievable with superchargers. We are considering what is just about practical for market entry, given that “fast” chargers typically take 3 hours to charge and the higher capacity Li-air battery would take correspondingly longer, we settle on 4 hours as a maximum charging time.

Theoretical energy density of chemistries

Comparison between conversion and intercalation chemistries becomes nontrivial due to their very different operating voltages and the need for support material in conversion chemistries.

To make the comparison shown here we consider each material vs some reference material, chosen to be at 1.5V and having no mass. This leads to its specific energy being defined as:

$$Specific\ energy = V_{Li^+\ vs\ ref} \cdot Specific\ capacity \qquad (1)$$

However one must take the magnitude of the voltage vs the reference electrode.

$$e_{cell} = \sum_{materials} \frac{M_{material}}{M_{total}} \cdot e_{material} \qquad (2)$$

Table 1 Average voltage of different materials and their capacity, effective Wh/kg vs 1.5V reference shown, all Li atoms considered to reside on the cathode. It is noted that values quoted here may be currently unachievable on long-duration cycling but are included to show the upper limits on Li-ion technology. The thick line shows the cut-off between anode and cathode materials.

	Average Voltage vs Li	Capacity mAh/g	Wh/g (2s.f.)
LiC_6	0.1[7]	340	480
$Li_{15}Si_4$	0.1[8]	1857	2600
Li	0.0	3862	5800
$LiCoO_2$ (LCO)	4.0[9]	295	740
Li_2MnO_3 (LMO)	3.5 (estimated)[10]	521	1000
O_2 (assuming mass is always in the cell)	2.96[11]	1675	1900

Table 2 Theoretical, chemistry only, energy densities of various anode-cathode combinations

Anode	Cathode	Wh/kg (3s.f)
LiC_6	LCO	615
LiC_6	LMO	699
LiC_6	"Air"	807
$Li_{15}Si_4$	LCO	992
$Li_{15}Si_4$	LMO	1380
$Li_{15}Si_4$	"Air"	2520
Li	LCO	1100
Li	LMO	1610
Li	"Air"	3460

Data for Walden plot

Table 3 Data for Walden plot

Solvent	Log[η^{-1}(Pa.S)]	Log[σ^{-1} (m^2/mol/Ω)]	B.p.t (K)	Log[O_2 Diff. (m^2/s)]	Salt (Conc.)	Implied radius O_2 (nm)
PC:DME (1:1)[12]	3.00	-2.92		-8.55	$LiClO_4$ (1M)	7.80E-11
DME[13]	3.38	-2.74	358	-8.13	LiTFSI (1M)	7.02E-11
Diglyme[13]	3.01	-2.92	435	-8.52	LiTFSI (1M)	7.43E-11
Tetraglyme[13]	2.44		549	-8.92		5.01E-11
DMSO[14]	2.68	-2.96	462	-8.68	$LiClO_4$ (1M)	5.02E-11
C_6F_{14}	3.40		339	-7.73		3.00E-11
$C_{10}F_{18}$	2.88		415	-8.64		7.19E-11
ACN[14]	3.47	-3.52	355		$LiClO_4$ (1M)	
THF[15]	3.35		339			
H_2O[16]	3.05		373	-8.71	KCl	1.24E-10
			Average radius (excluding H_2O)			6.07E-11

References

1 P. Liu and S. Meng, *Battery500 Consortium: Development of High Capacity Cathodes and Robust Solid Electrolytes*, Univ. of California, San Diego, CA (United States), 2022.

2 D. J. C. MacKay, *Sustainable energy - without the hot air*, UIT Cambridge, Cambridge, repr., 2011.

3 B. Cárdenas, L. Swinfen-Styles, J. Rouse and S. D. Garvey, *Energies*, 2021, 14, 8524.

4 M. K. Singla, P. Nijhawan and A. S. Oberoi, *Environ Sci Pollut Res*, 2021, 28, 15607–15626.

5 Z. Yue and S. Som, *Applied Energy*, 2021, 281, 114221.

6 J. Laborda and M. J. Moral, *Transportation Research Part A: Policy and Practice*, 2019, 124, 488–504.

7 S. S. Zhang, *J. Electrochem. Soc.*, 2020, 167, 100510.

8 M. T. McDowell, S. W. Lee, W. D. Nix and Y. Cui, *Adv. Mater.*, 2013, 25, 4966–4985.

9 J. Li, C. Lin, M. Weng, Y. Qiu, P. Chen, K. Yang, W. Huang, Y. Hong, J. Li, M. Zhang, C. Dong, W. Zhao, Z. Xu, X. Wang, K. Xu, J. Sun and F. Pan, *Nat. Nanotechnol.*, 2021, 16, 599–605.

10 F. Susai, M. Talianker, J. Liu, Rosy, T. Paul, Y. Grinblat, E. Erickson, M. Noked, L. Burstein, A. Frenkel, Y. Tsur, B. Markovsky and D. Aurbach, *Materials (Basel, Switzerland)*, , DOI:10.3390/ma13194388.

11 W.-J. Kwak, Rosy, D. Sharon, C. Xia, H. Kim, L. R. Johnson, P. G. Bruce, L. F. Nazar, Y.-K. Sun, A. A. Frimer, M. Noked, S. A. Freunberger and D. Aurbach, *Chemical Reviews*, , DOI:10.1021/acs.chemrev.9b00609.

12 G. Cookey, *IOSR Journal of Applied Chemistry (IOSR-JAC)*, 2015, 8, 35–41.

13 A. Schürmann, R. Haas, M. Murat, N. Kuritz, M. Balaish, Y. Ein-Eli, J. Janek, A. Natan and D. Schröder, *Journal of The Electrochemical Society*, 2018, 165, A3095–A3099.

14 N. Mozhzhukhina, M. P. Longinotti, H. R. Corti and E. J. Calvo, *Electrochimica Acta*, 2015, 154, 456–461.

15 M. Morita, F. Tachihara and Y. Matsuda, *Electrochimica Acta*, 1987, 32, 299–305.

16 P. Han and D. M. Bartels, *J. Phys. Chem.*, 1996, 100, 5597–5602.
